# *In silico* analysis and preclinical findings uncover potential targets of anti-cervical carcinoma and COVID-19 in laminarin, a promising nutraceutical

**DOI:** 10.3389/fphar.2022.955482

**Published:** 2022-08-09

**Authors:** Jiaqi Liu, Yudong Chen, Litao Nie, Xiao Liang, Wenjun Huang, Rong Li

**Affiliations:** ^1^ Key Laboratory of Environmental Pollution and Integrative Omics, Guilin Medical University, Education Department of Guangxi Zhuang Autonomous Region, Guilin, China; ^2^ Department of Gynecology, Guigang City People’s Hospital, The Eighth Affiliated Hospital of Guangxi Medical University, Guigang, China; ^3^ Key Laboratory of Tumor Immunology and Microenvironmental Regulation, Guilin Medical University, Guilin, China

**Keywords:** functional foods, laminarin, cervical carcinoma, COVID-19, biotargets

## Abstract

Until today, the coronavirus disease 2019 (COVID-19) pandemic has caused 6,043,094 deaths worldwide, and most of the mortality cases have been related to patients with long-term diseases, especially cancer. Autophagy is a cellular process for material degradation. Recently, studies demonstrated the association of autophagy with cancer development and immune disorder, suggesting autophagy as a possible target for cancer and immune therapy. Laminarin is a polysaccharide commonly found in brown algae and has been reported to have pharmaceutic roles in treating human diseases, including cancers. In the present report, we applied network pharmacology with systematic bioinformatic analysis, including gene ontology (GO) enrichment, Kyoto Encyclopedia of Genes and Genomes (KEGG) enrichment analysis, reactome pathway analysis, and molecular docking to determine the pharmaceutic targets of laminarin against COVID-19 and cervical cancer *via* the autophagic process. Our results showed that the laminarin would target ten genes: *CASP8*, *CFTR*, *DNMT1*, *HPSE*, *KCNH2*, *PIK3CA*, *PIK3R1*, *SERPINE1*, *TLR4*, and *VEGFA*. The enrichment analysis suggested their involvement in cell death, immune responses, apoptosis, and viral infection. In addition, molecular docking further demonstrated the direct binding of laminarin to its target proteins, VEGFA, TLR4, CASP8, and PIK3R1. The present findings provide evidence that laminarin could be used as a combined therapy for treating patients with COVID-19 and cervical cancer.

## Introduction

Since the outbreak of COVID-19 in 2019, there have been over 456,797,217 confirmed cases of COVID-19 worldwide, leading to 6,043,094 deaths, as reported to the World Health Organization (WHO) (14 March 2022). The mortality rate of the latest variant, omicron, is relatively low, but its high infection rate continues to be a pressing problem in our society (Bouzid et al., 2022). Moreover, the mortality rate is very high in patients with long-term diseases, especially cancer patients (Aghamirza Moghim Aliabadi et al., 2022). Therefore, there is an urgent need to identify alternative drug or compound to help patients with COVID-19 and cancer. Cervical carcinoma is a common malignant tumor that occurs in the female reproductive system (Cohen et al., 2019). According to the data from the WHO, cervical carcinoma is the fourth most common cancer among women, leading to 342,000 deaths in 2020 globally (https://www.who.int/news-room/fact-sheets/detail/cervical-cancer).

Autophagy is a mechanism for cellular material degradation that occurs under stressful conditions (Yun and Lee, 2018). Cumulating studies showed that autophagy disorder is closely associated with the development of cancer (Levy et al., 2017). In cervical cancer, autophagy-related genes were reported to predict the prognosis of cervical cancer (Jiang et al., 2021). *In vitro* and *in vivo* protective autophagic mechanisms resulted in antitumor activity in cervical cancer (Zhang F. et al., 2022). On the contrary, autophagy was found to interplay with the antiviral IFN-I response, suggesting the targeting of autophagy as a potential target and strategy for treating COVID-19 (Bonam et al., 2020; García-Pérez et al., 2020). Therefore, targeting autophagy could be a potential option for treating patients with COVID-19 and cervical cancer.

Laminarin is a polysaccharide commonly found in brown algae (Chen et al., 2021). Recent studies suggested the pharmaceutic roles of laminarin in human diseases, including cancers. A study of colorectal carcinoma cells demonstrated the combined anticancer effect of sulfated laminarin (Malyarenko et al., 2021). Zhang’s group also suggested that laminarin sulfate can be used as a carrier to inhibit heparinase and treat melanoma lung metastasis (Zhang et al., 2021). Besides treating cancer, laminarin was reported to regulate IL-10 production and host immunogenicity (Ferreira et al., 2007; Korotchenko et al., 2021). In this report, we applied network pharmacology with bioinformatics analysis, including gene ontology (GO) enrichment, Kyoto Encyclopedia of Genes and Genomes (KEGG) enrichment analysis, and molecular docking, to investigate the pharmaceutic targets of laminarin against COVID-19 and cervical cancer through autophagy. The findings of the present study provide evidence that laminarin could be used as a combined therapy for treating patients with COVID-19 and cervical cancer.

## Materials and methods

### Identification of laminarin-, cervical carcinoma-, COVID-19-, and autophagy-associated genes

The mRNA expression profile of cervical carcinoma (CC) was obtained upon searching the dataset of GSE63514 from the Gene Expression Omnibus (GEO) database, which included 24 normal and 28 cervical cancers (Barrett et al., 2013). The differentially expressed genes (DEGs) of cervical carcinoma were analyzed by the GEO2R tool (den Boon et al., 2015). The genes with |log_2_ fold change| > 1 and −log_10_ *p*-value > 1.3 were considered DEGs. For the identification of COVID-19- and autophagy-associated genes, the online databases including the GeneCards database (Stelzer et al., 2016), Online Mendelian Inheritance in Man (OMIM) database (Hamosh et al., 2002), and National Center for Biotechnology Information (NCBI) were accessed (National Library of Medicine National Center for Biotechnology Information (NCBI), 2022). The pharmaceutical targets of laminarin were harvested using online databases, including Comparative Toxicogenomics Database (CTD) (Davis et al., 2020), Swiss Target Prediction database (Daina et al., 2019), SuperPred database (Nickel et al., 2014), and PharmMapper (Wang X. et al., 2017). The target genes were subjected to UniProt Knowledgebase (UniProtKB) for human database correction (The UniProt Consortium, 2021). To further determine the targets of laminarin against cervical carcinoma and COVID-19 through autophagy, the laminarin-, CC-, COVID-19- and autophagy-associated genes and targets were overlapped. The common genes were considered the possible targets.

### Core target screening and network analysis

The common genes were subjected to Bioconductor packages, including ClusterProfiler and ggplot2 tools, to analyze and visualize the biological processes and pathways controlled by the targets of laminarin against cervical carcinoma and COVID-19 through autophagy. ClusterProfiler provides GO enrichment, KEGG enrichment analysis, and Reactome pathway analysis (Jassal et al., 2020; Wu T. et al., 2021). Ggplot2 was used to visualize the enrichment results. The terms with adjusted *p*-value < 0.05 was considered statistically significant. The targets were used for protein–protein interaction (PPI) analysis using the STRING platform, and the confidence score was 0.4 (Szklarczyk et al., 2021). The NetworkAnalyzer of Cytoscape software was also used to integrate the result of targets identification, GO, and KEGG (Shannon et al., 2003).

### Molecular docking

The binding affinity of laminarin with its predicted targets was assessed using molecular docking. The chemical structure of laminarin was obtained from the PubChem database (Wang Y. et al., 2017). The protein structure of VEGFA, TLR4, and CASP8 was obtained from the Protein Data Bank (PDB) (Berman et al., 2000). The ChemBio3D Draw tool of ChemBioOffice 2010 software was used to download three-dimensional structures of these proteins, followed by the MM2 energy optimization. The PDB file was converted to the pdbqt file format using AutoDock Tools 1.5.6 of the AutoDock Vina software (that can be recognized by the AutoDock Vina program for ligand docking program) (Trott and Olson, 2010). The docking parameter was determined according to the size of the root-mean-square deviation (RMSD) between the docked ligand molecule and the original ligand molecule. An RMSD of less than 4 Å was the threshold for the conformation of the ligand for matching the conformation of the original ligand in the molecular docking analysis.

## Results

### Identification of laminarin’s targets against cervical carcinoma and COVID-19 through autophagy

**FIGURE 1:**
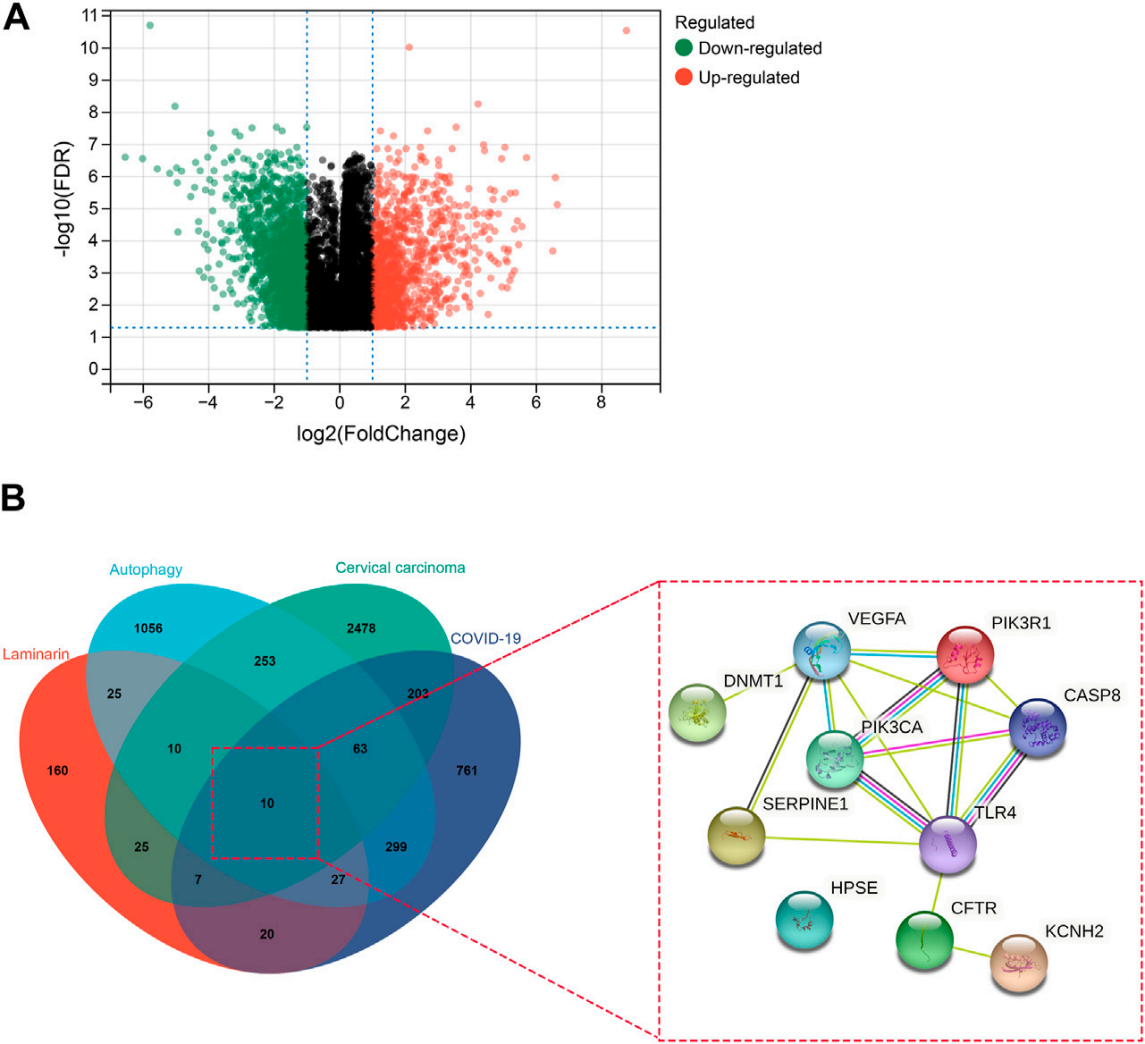
Identification of laminarin’s targets against cervical carcinoma and COVID-19 through autophagy. **(A)** Volcano plot showed the differential gene expression in cervical carcinoma. Genes with |log_2_ fold change| > 1 and −log_10_ *p*-value > 1.3 were considered differentially expressed genes. Green dots represented downregulated genes, and red dots represented upregulated genes. **(B)** Left panel: Venn diagram showed the number of overlapped laminarin-, COVID-19-, cervical carcinoma-, and autophagy-associated genes. Right panel: protein–protein interaction of the laminarin-, COVID-19-, cervical carcinoma-, and autophagy-associated genes.

Upon analysis of the GSE63514 dataset of GEO, we identified 3,169 DEGs, including 1,093 upregulated genes and 2,076 downregulated genes in cervical carcinoma (Figure 1A). By searching the databases, we obtained 1,390 COVID-19-associated genes (Figure 1B). In addition, we found 284 pharmaceutical targets of laminarin and 1,744 autophagy-associated genes (Figure 1B). The identified gene and targets were overlapped to determine the possible targets of laminarin against cervical carcinoma and COVID-19 through autophagy. Ten common targets (CASP8, CFTR, DNMT1, HPSE, KCNH2, PIK3CA, PIK3R1, SERPINE1, TLR4, and VEGFA) were observed, and the PPI analysis showed their interaction (Figure 1B).

### Laminarin’s targets were involved in the cell death and immune responses

**FIGURE 2:**
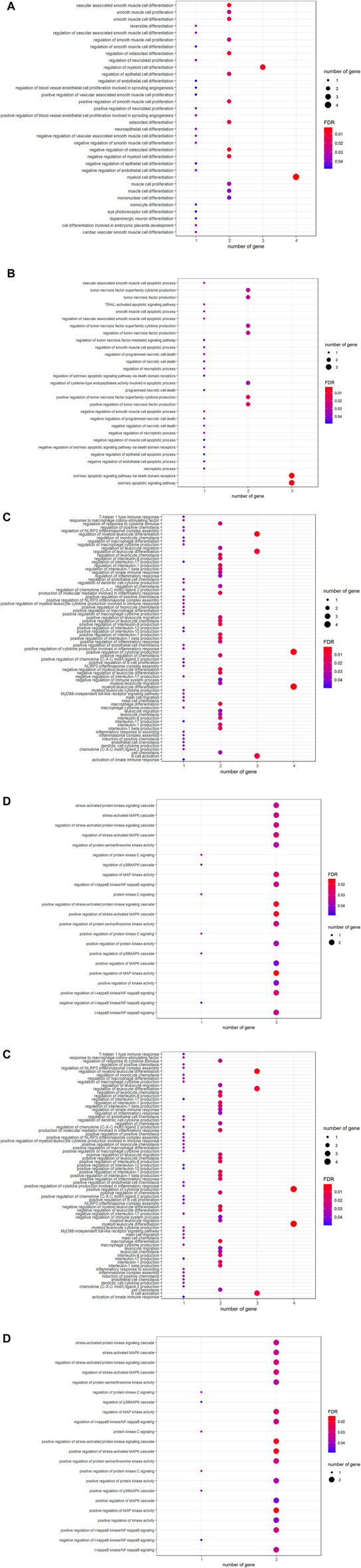
Involvement of laminarin’s targets in cell death and immune responses. Gene ontology enrichment analysis of laminarin’s targets highlighted the biological processes related to **(A)** cell proliferation and differentiation, **(B)** cell death, **(C)** immune responses, and **(D)** kinase activities. **(E)** Laminarin’s targets contributed to molecular functions, including binding and ion channel activities. **(F)** Laminarin’s targets played roles in different cellular components. The size of a bubble represents the number of genes, and the color of the bubble represents the significance of the terms.

To understand the biological roles controlled by laminarin targets, GO enrichment analysis was applied. The result of GO analysis highlighted the biological processes related to cell proliferation and differentiation (Figure 2A), cell death such as extrinsic apoptotic signaling pathway, TRAIL-activated apoptotic signaling pathway, and regulation of necroptotic process (Figure 2B). In addition, laminarin’s targets played roles in immune responses such as regulation of innate immune response, regulation of leukocyte differentiation, macrophage differentiation, B-cell activation, and production of interleukin (Figure 2C). Furthermore, many kinase activities such as regulation of MAP kinase activity, regulation of I-kappaB kinase/NF-kappaB signaling, regulation of p38MAPK cascade, and protein kinase C signaling were highlighted in our result (Figure 2D). In terms of molecular function, laminarin’s targets contributed to different binding and ion channel activities, especially the functions related to immune functions such as cytokine receptor binding, tumor necrosis factor receptor binding, and chemoattractant activity (Figure 2E). These biological processes and molecular functions were suggested to occur in different cellular components, including platelet alpha granule lumen, ion channel complex, cytoplasmic vesicle lumen, perinuclear endoplasmic reticulum, pericentric heterochromatin, transmembrane transporter complex, transporter complex, early endosome, phagocytic cup, and phosphatidylinositol 3-kinase complex (Figure 2F).

### Laminarin’s targets contributed to the pathways related to cancer and viral infection

**FIGURE 3:**
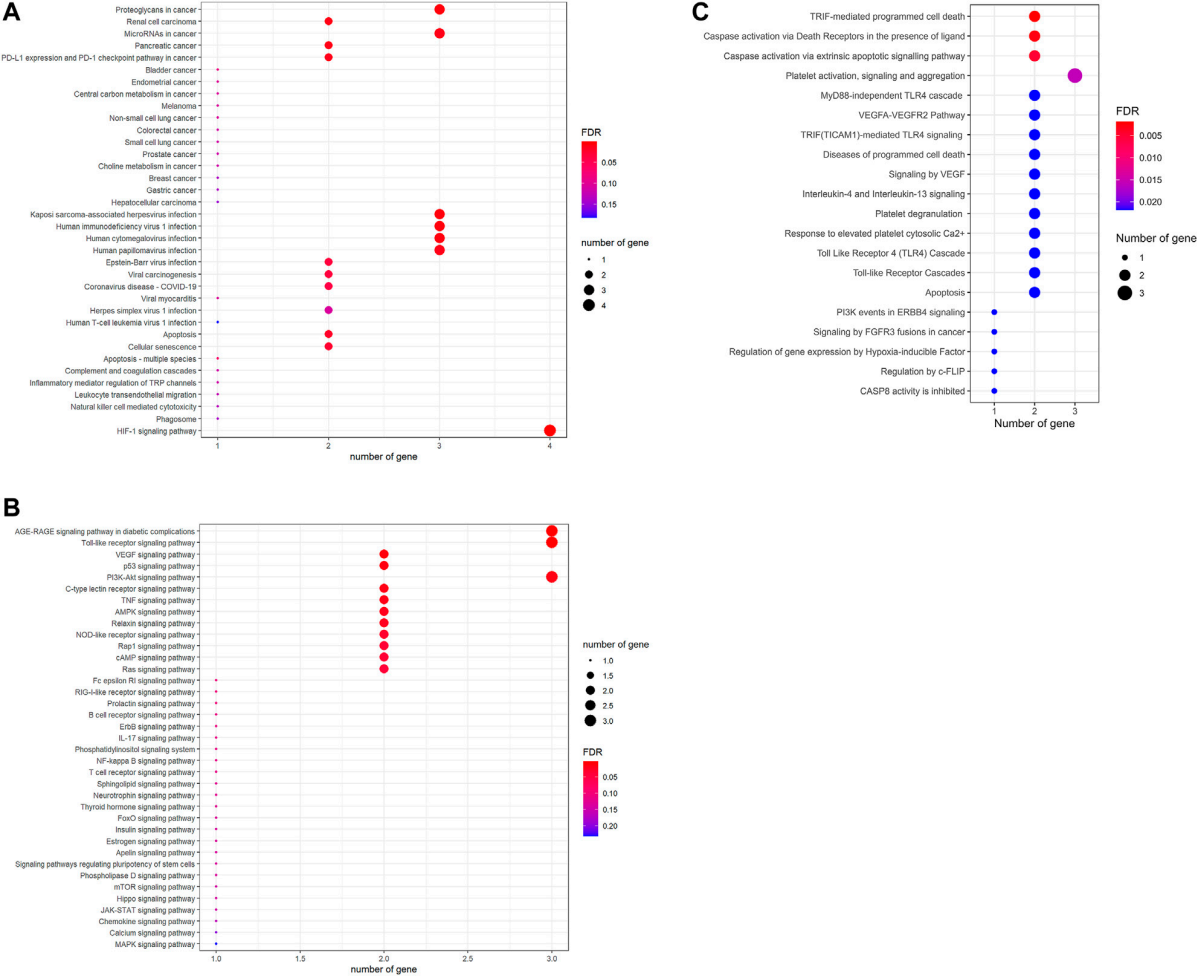
Laminarin’s targets involved in pathways related to cancer and cell apoptosis. Kyoto Encyclopedia of Genes and Genomes pathway enrichment analysis highlighted the involvement of laminarin’s targets in **(A)** cancer, viral infection, cell death, and immune responses; and **(B)** cell signaling pathways related to cancer development. **(C)** Reactome pathway analysis highlighted the importance of laminarin in cell apoptosis. The size of a bubble represents the number of genes, and the color of the bubble represents the significance of the pathways.

To further investigate the signaling pathways controlled by laminarin’s targets, KEGG enrichment analysis, and Reactome pathway analysis were conducted. The result of the KEGG enrichment analysis showed the involvement of laminarin’s targets in cancer, viral infection, cell death, and immune responses (Figure 3A). More importantly, many cell signaling pathways such as Toll-like receptor signaling pathway, VEGF signaling pathway, p53 signaling pathway, PI3K-Akt signaling pathway, TNF signaling pathway, AMPK signaling pathway, NOD-like receptor signaling pathway, Rap1 signaling pathway, FoxO signaling pathway, mTOR signaling pathway, Hippo signaling pathway, JAK-STAT signaling pathway, and MAPK signaling pathway related to carcinogenicity and cell proliferation were highlighted in our result (Figure 3B). In the Reactome pathway analysis, our result highlighted the contribution of laminarin to cell apoptosis through the regulation of TRIF-mediated programmed cell death and caspase activation *via* death receptors and extrinsic apoptotic signaling pathway (Figure 3C). Taken together, our results suggested that the laminarin targets played important roles in carcinogenicity and cell apoptosis.

### Direct binding of laminarin to its target proteins, VEGFA, TLR4, CASP8, and PIK3R1

**FIGURE 4:**
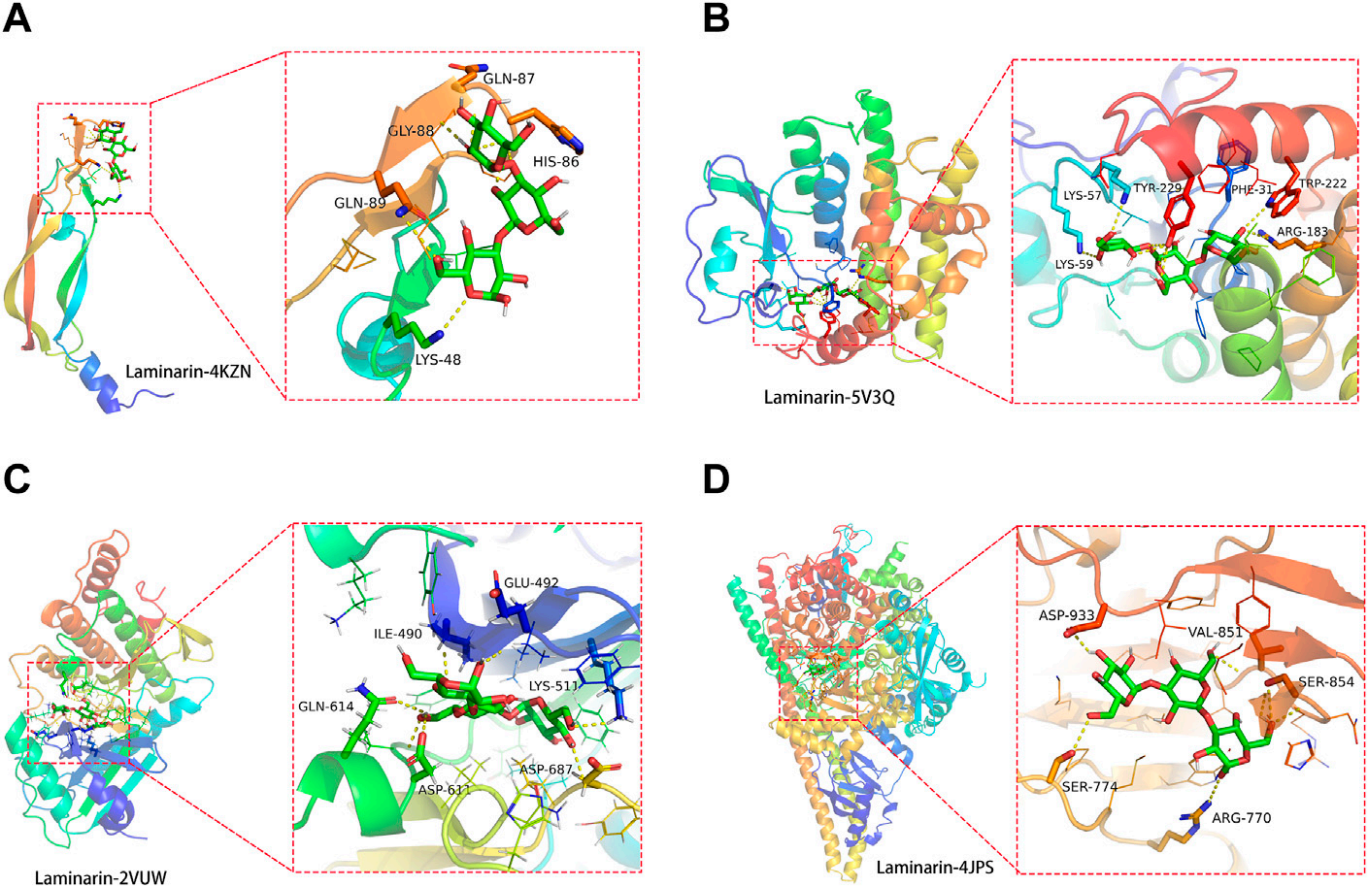
Direct binding of laminarin with its target proteins. Molecular docking analysis showed the hydrogen bond formation between laminarin with the amino residues of **(A)** VEGFA (ID: 4KZN), **(B)** TLR4 (ID: 5V3Q), **(C)** CASP8 (ID: 2VUW), and **(D)** PIK3R1 (ID: 4JPS).

Before conducting the molecular docking analysis, laminarin’s targets were prioritized using the Cytoscape_v3.8.2 tool. Four core targets of laminarin (VEGFA, TLR4, CASP8, and PIK3R1) were obtained under the setting with a medium of freedom of 2.5 and the highest freedom of 5 (Figure 4A). The protein structures of VEGFA (ID: 4KZN) (Shaik et al., 2020), TLR4 (ID: 5V3Q) (Menon et al., 2017), CASP8 (ID: 2VUW) (Eswaran et al., 2009), and PIK3R1 (ID: 4JPS) (Furet et al., 2013) were obtained from the PDB database. Then, these protein structures were subjected to molecular docking analysis with laminarin using the AutoDock Vina program, and the results were displayed using PyMOL (version 2.3). Our results showed the possible direct binding of laminarin to these four target proteins, which was reflected by the negative value of the binding affinity (Figure 4). We found the formation of hydrogen bonds between laminarin with LYS-48 (3.2Å), HIS-86 (3.2 Å), GLN-87 (3.3Å), GLY-88 (2.2Å), and GLN-89 (3.4 Å) of VEGFA (ID: 4KZN) (Figure 4A). The binding affinity was −4.2 kcal/mol. For the TLR4 (ID: 5V3Q), its amino acid residues LYS-59 (3.1 Å), LYS-57 (2.8 Å), TYR-229 (3.0Å), PHE-31 (2.3 Å), ARG-183 (3.3 Å), and TYP-222 (3.0Å) formed hydrogen bonds with laminarin, and the binding affinity was −6.2 kcal/mol (Figure 4B). Similar bindings were observed between laminarin and CASP8 (ID: 2VUW) through the amino acid residues of ASP-687 (2.7 Å), GLU-492 (2.2 Å), GLN-614 (2.7 Å), LYS-511 (2.5Å), ILE-490 (2.4 Å), and ASP-611 (2.2 Å) with −8.4 kcal/mol (Figure 4C). In addition, laminarin formed hydrogen bonds with amino acid residues, ASP-933 (2.3Å), SER-774 (2.9 Å), ARG-770 (3.3Å), SER-854 (3.0Å), and VAL-851 (2.8 Å) of PIK3R1 (ID: 4JPS) (Figure 4D). The binding affinity was −6.9 kcal/mol. Taken together, our data suggested that laminarin could directly bind to its target proteins.

## Discussion

**FIGURE 5:**
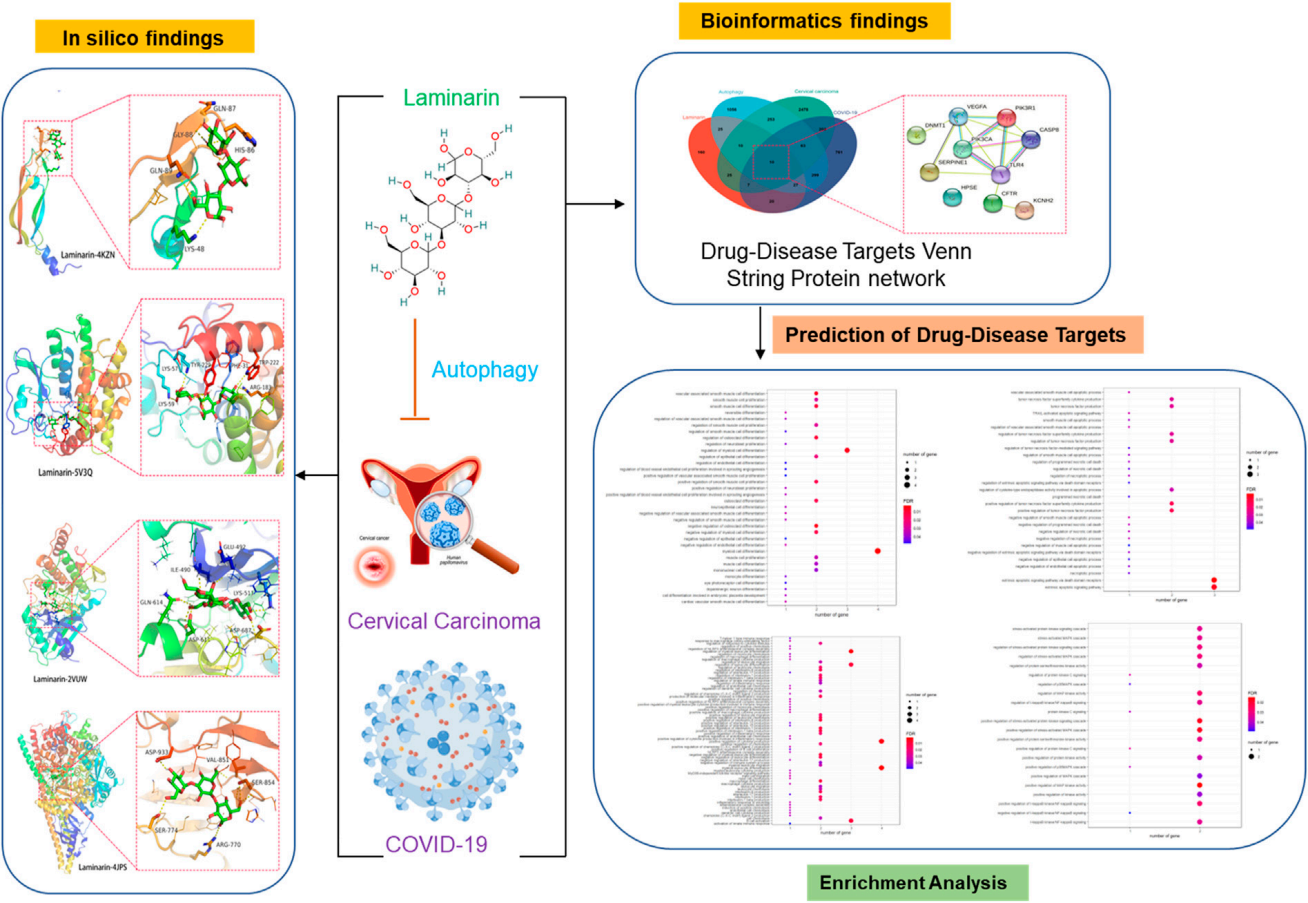
Workflow of the study. The pharmaceutic targets and their function roles were identified using network pharmacology, gene ontology enrichment analysis, and molecular docking.

This study aimed to investigate the possible use of laminarin for treating cervical carcinoma and COVID-19 by targeting autophagy (Figure 5). By searching different databases, we identified a cluster of common associated genes of laminarin, cervical carcinoma, COVID-19, and autophagy. The following genes were considered the possible targets of laminarin against cervical carcinoma and COVID-19 *via* autophagy: *CASP8*, *CFTR*, *DNMT1*, *HPSE*, *KCNH2*, *PIK3CA*, *PIK3R1*, *SERPINE1*, *TLR4*, and *VEGFA*. Bioinformatic analysis, including network pharmacology, GO enrichment analysis, and molecular docking, was further used to characterize the biological functions and the underlying mechanisms controlled by these genes. The result of GO highlighted the involvement of these genes in cell death and immune responses, suggesting that laminarin could mediate autophagic cell death. Autophagy plays a dual role as a proviral and an antiviral factor during virus replication and is considered a target for developing an effective anti-SARS-CoV-2 treatment (Liang et al., 2021). Some reports demonstrated the close association between autophagy and SARS-CoV-2. A kinase phosphorylation study showed that SARS-CoV-2 regulated various signaling pathways, including autophagy (Chatterjee and Thakur, 2022). A genome-wide CRISPR/Cas-9 knockout (KO) screening suggested that autophagy-related genes were required for SARS-CoV-2 replication (Kratzel et al., 2021). More importantly, studies found that induction of autophagy is essential for the infection and replication of SARS-CoV-2 through the regulation of acute inflammatory responses (Zhang X. et al., 2022). On the contrary, autophagy also played an important role in cervical cancer. It was reported that autophagy controlled the epithelial-to-mesenchymal transition and metastasis of cervical cancer through the regulation of the NOTCH1 intracellular domain (Zada et al., 2022). In addition, autophagy was crucial for the sensitivity of cervical cancer cells to cisplatin chemotherapy (Huang et al., 2021). The application of laminarin to therefore target autophagy could be an alternative strategy for patients with COVID-19 and cervical carcinoma.

In the pathway analysis, we found that laminarin’s targets played a role in many cell signaling pathways related to COVID-19 and cervical carcinoma. The VEGF signaling pathway plays a vital role in the pathologies of COVID-19-associated pulmonary edema, sepsis, and acute lung injury (Wang et al., 2021). A clinical comparison study among the COVID-19 patient categories found a significantly lower level of VEGF in the mildly symptomatic patients (Tripathy et al., 2021). In addition, VEGF blockade and antagonist were reported to be helpful in the treatment of COVID-19 (Sahebnasagh et al., 2021; Lampropoulou et al., 2022). In cervical cancer, VEGF signaling regulated several oncogenic signaling pathways and cancer-regulated angiogenesis (Prasad et al., 2022). Functionally, VEGF controlled proliferation and apoptosis in cervical cancer. (Wu X. et al., 2021). Hence, VEGF was considered a therapeutic target for recurrent and metastatic cervical cancer (Chuai et al., 2021). The VEGF inhibitor bevacizumab was reportedly used in advanced metastatic cervical cancer (Singh et al., 2022). Besides VEGF signaling, we also found the regulation of the p53 signaling pathway by laminarin’s targets. This pathway was reported to be associated with COVID-19 infection. A clinical study demonstrated that peripheral blood mononuclear cells of COVID-19 patients had a higher p53 expression, which positively correlated with the level of inflammatory cytokines (Bordoni et al., 2021). In addition, p53 signaling is related to immune and apoptosis-related functions of SARS-CoV-2 infected cells (Rahaman et al., 2021) because SARS-CoV-2 could favor tumor growth by inducing MDM2-mediated p53 downregulation (Tanda et al., 2022) and target mTOR and RPS6KB1 to inhibit viral replication in the human respiratory tract and lung cells (Ramaiah, 2020). Furthermore, single-nucleotide polymorphism of p53 played a role in the innate immune response and restricted the SARS-CoV-2-mediated mortality rate (Lodhi et al., 2021). By targeting these signaling pathways, laminarin could be a potential compound to treat patients with cervical cancer and COVID-19.

For the latter part, we conducted a molecular docking study to investigate the possible direct binding of laminarin to its core target proteins. Molecular docking is a key tool commonly used to predict the predominant binding of a ligand with a protein of known three-dimensional structure (Morris and Lim-Wilby, 2008). Our result showed that laminarin had a good binding affinity to VEGFA, TLR4, CASP8, and PIK3R1. Vascular endothelial growth factor A (VEGFA) is involved in the VEGF signaling pathway, and we have discussed its roles in COVID-19 and cervical cancer above. Toll-like receptor 4 (TLR4) played an important role in the generation of an antiviral state after being infected with pathogenic viruses (Boodhoo et al., 2022). A study of the human pluripotent stem cell–based model of SARS-CoV-2 infection demonstrated the involvement of TLR4 in the inflammatory activation of vascular endothelial cells (Ma et al., 2022), and TLR4 was reported to contribute to anti-inflammatory effects and SARS-CoV-2 infection *in vitro* (Numata and Voelker, 2022). Therefore, targeting the TLR4 inflammatory pathway could serve as a potential strategy in reducing inflammatory lung injury (Bermudez et al., 2022). Caspase 8 (CASP8) is a key regulator of extrinsic apoptosis and necroptosis pathways (Tummers and Green, 2017), which is associated with COVID-19 infection. A study of 101 plasma proteins of hospitalized COVID-19 patients demonstrated a positive correlation between inflammatory markers and CASP8 (Haljasmägi et al., 2020). More importantly, SARS-CoV-2 infection activated CASP8 to trigger cell apoptosis and inflammatory cytokine processing in the lung epithelial cells (Li et al., 2020), suggesting CASP8 to be a target to relieve the severity of SARS-CoV-2 infection.

In conclusion, our data, for the first time, provide evidence that laminarin could be an alternative treatment for patients with cervical cancer and COVID-19 by targeting autophagy. However, as our findings were drawn from network pharmacology, further clinical verification and molecular functional characterization are needed to warrant the present findings from bioinformatic analysis before clinical use of laminarin.

## Data Availability

The original contributions presented in the study are included in the article/Supplementary Material. Further inquiries can be directed to the corresponding authors.
